# Diversity-Oriented Synthesis: Amino Acetophenones as Building Blocks for the Synthesis of Natural Product Analogs

**DOI:** 10.3390/ph14111127

**Published:** 2021-11-05

**Authors:** Mathias Eymery, Viet-Khoa Tran-Nguyen, Ahcène Boumendjel

**Affiliations:** 1 Université Grenoble Alpes, INSERM, LRB, 38000 Grenoble, France; meymery@embl.fr; 2 EMBL Grenoble, 71 Avenue des Martyrs, CS 90181, 38042 Grenoble, France; 3 Laboratoire d’Innovation Thérapeutique, Université de Strasbourg, 67400 Illkirch, France; khoatnv1993@gmail.com

**Keywords:** Diversity-Oriented Synthesis, natural products, amino acetophenones, flavones, aurones, coumarins, quinolones, azocanes

## Abstract

Diversity-Oriented Synthesis (DOS) represents a strategy to obtain molecule libraries with diverse structural features starting from one common compound in limited steps of synthesis. During the last two decades, DOS has become an unmissable strategy in organic synthesis and is fully integrated in various drug discovery processes. On the other hand, natural products with multiple relevant pharmacological properties have been extensively investigated as scaffolds for ligand-based drug design. In this article, we report the amino dimethoxyacetophenones that can be easily synthesized and scaled up from the commercially available 3,5-dimethoxyaniline as valuable starting blocks for the DOS of natural product analogs. More focus is placed on the synthesis of analogs of flavones, coumarins, azocanes, chalcones, and aurones, which are frequently studied as lead compounds in drug discovery.

## 1. Introduction

Due to an increasing and incessant need for small molecules used as drug candidates or molecular tools for therapeutic applications, organic and medicinal chemistry is considered the crux of both chemical genetics and drug discovery [1,2]. The main strategies to synthesize chemically diverse molecules can be classified into three approaches, namely, Target-Oriented Synthesis (TOS), Combinatorial Synthesis (CS), and Diversity-Oriented Synthesis (DOS). The TOS approach is focused on the synthesis of a single compound or a restricted class of compounds directed towards a biological target for a specific pharmaceutical area. TOS is the oldest approach whose advantage is the possibility to be planned through retrosynthetic analysis [3]. CS, on the other hand, has emerged as an indispensable strategy to significantly increase the diversity as well as the number of small molecules to be used for therapeutic and biological applications, contributing to the rapid discovery of potential hits [3]. Thanks to solid phase organic synthesis, which has its origins in peptide synthesis, CS takes full advantage of available automated systems. However, disadvantages may stem from the library design involved in this approach. Finally, DOS established itself as an attractive approach to produce large-sized chemical libraries [3,4], allowing access to a high number of compounds in a few steps starting from one building block that has to be affordable, safe, and either commercially available or easily prepared on a large scale [5,6,7,8,9]. On the basis of comparing the three aforementioned approaches, it can be deduced that TOS and CS focus on a few points in the chemical space, whereas DOS is meant to ensure large coverage of both the chemical space and molecular diversity, which is a significant advantage.

Besides the synthetic chemistry strategies discussed above, drug repositioning and natural products may lead to the discovery of potential hits. Drug repositioning, using large libraries of clinically used drugs to supplement high-throughput screening [10], is widely considered a promising approach as it speeds up the drug discovery process by bypassing some clinical trials that usually take up too much time and massive financial support. The natural products arena, on the other hand, is another major source of compounds from different origins, including plants, fungi, algae, insects, and animals [11,12], with countless known molecules and analogs used as major drugs targeting life-threatening diseases. In addition to relevant biological effects, natural products offer extraordinary and unpredictable chemical diversity that constitutes a source of inspiration for the medicinal chemistry community.

Based on the importance of both DOS and natural products in drug discovery, their combination could facilitate and accelerate the identification of novel compounds with improved biological activities [13,14]. This article, for this reason reviews the DOS of natural product analogs with a special focus on the synthesis of analogs of flavonoids and coumarins.

Flavonoids are chemical entities commonly found in fruits and vegetables. In addition to their contribution to plant growth and defense, they exhibit a wide range of biological effects against major diseases. The most known activity of flavonoids is their antioxidant effect, and, for this fact, they are often considered unique allies for the prevention of cardiovascular diseases, cancer, and neurological disorders [15,16,17]. Based on their structures, flavonoids are classified into many different sub-classes including flavones, isoflavones, flavonols, flavanones, chalcones, and aurones. In drug discovery, the tricyclic structure of naturally occurring flavonoids (2-Phenyl-4*H*-1-benzopyran-4-one) is used as a scaffold in pharmacophore-based drug design. The introduction of diverse functions on this chemotype has led to the identification of promising leads and drug candidates. One of the structural modifications operated during the optimization process is the introduction of amino groups onto the scaffold, either at the periphery of the flavonoidic structure (aminoflavones) or at the central ring through the replacement of the oxygen by an amino group (turning flavones to 2-phenyl-4-quinolones) [18,19]. In the same vein, the naturally occurring coumarins (1-benzopyran-2-one) were extensively investigated as scaffolds in drug discovery [20]. Similarly to what was said for flavonoids, one of the chemical modifications operated on coumarins is the substitution of the furanone ring’s oxygen atom with an amino group (turning coumarins into 2-quinolones). Classically, the introduction of amines on the flavonoid and the coumarin skeletons requires several synthetic steps and, in some cases, the use of toxic reagents [21]. This is especially the case when multifunctional derivatives of flavones and coumarins are targeted.

**Figure 1:**
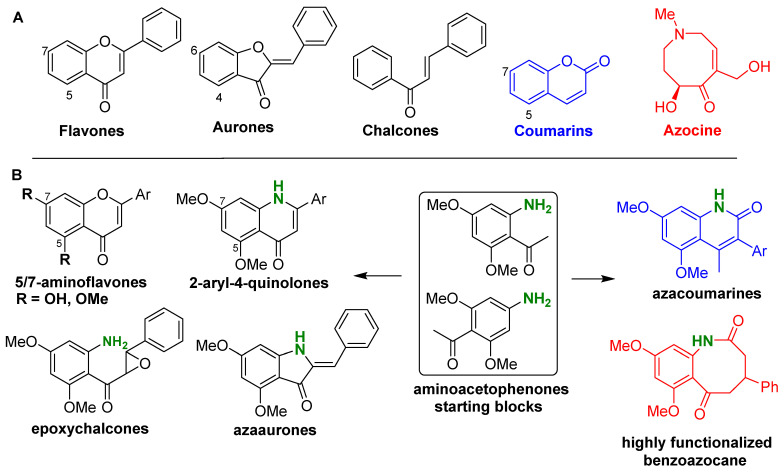
Chemical structures reported in this review. (**A**) General structures of the targeted natural products; (**B**) Structures of the starting blocks and natural product analogs prepared.

Based on our own work and literature survey, it was found that amino dimethoxyacetophenones can be used as a “Swiss army knife” that allows the access to a wide variety of bioactive compounds derived from flavones, aurones, chalcones, and coumarins in which an amino group was introduced (Figure 1). This includes aminoflavones, quinolones, azaaurones, aminoepoxychalcones, and azacoumarines. Moreover, by using the same agents, a highly functionalized azocine derivative, a benzoazocane recognized for its diverse biological activities, can be prepared through a novel and short synthetic scheme (Figure 1). It should be mentioned that the use of aminoacetophenone in drug discovery is ancient, as exemplified by chloramphenicol that is a widely produced antibiotic in recent decades [22].

As points taken in the present review, we summarize and discuss different methods for the synthesis of 5-aminoflavones, 7-aminoflavones, 2-aryl-4-quinolones, 3-aryl-2-quinolones, azaaurones, and a benzoazocane starting from 2-amino-4,6-dimethoxyacetophenone and 4-amino-2,6-dimethoxyacetophenone.

## 2. Synthesis of the Starting Blocks

**Scheme 1:**
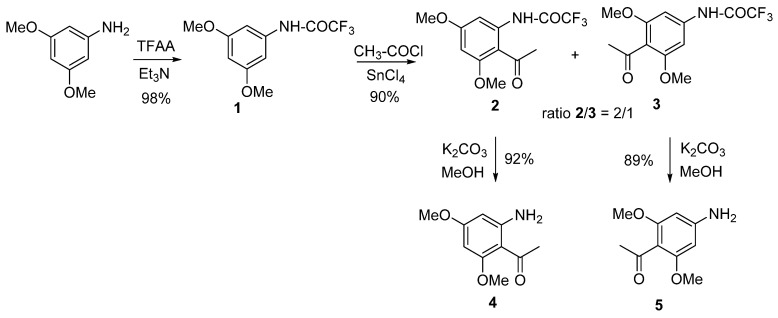
Synthesis of the starting blocks.

The aminodimethoxyacetophenones used as starting blocks were 2-amino-4,6-dimethoxyacetophenone (**4**) and 4-amino-2,6-dimethoxyacetophenone (**5**) (Scheme 1). The synthesis pathways of **4** and **5** are displayed in Scheme 1. The presence and the positions of the methoxy groups on the starting blocks were chosen in line with the common substitution patterns found in naturally occurring flavones, aurones, and coumarins, over 50% of which are hydroxylated and/or methoxylated at the 5, 7 positions (in flavonoids) and the 4, 6 positions (in aurones).

The synthesis of the starting blocks was carried out from the commercially available 3,5-dimethoxy aniline, which was protected as a trifluoroacetamide (compound **1**) then reacted with acetyl chloride in the presence of tin tetrachloride (SnCl_4_) to afford the two acetylated regioisomers **2** and **3** with a ratio of 2/1, respectively. The best Lewis acid needed for the acetylation step by far was SnCl_4_ (compared to AlCl_3_, SnCl_2_, ZnCl_2_), as it allowed the reaction to proceed in smooth conditions [23]. The trifluoroacetamide was deprotected in classical conditions by means of potassium carbonate in methanol to afford the corresponding aminoacetophenones **4** and **5** with excellent yields.

During the synthesis of **2** and **3**, it was reported that, in the presence of a large excess of SnCl_4_, only the formation of **3** was observed, whereas **2** was only produced in traces. Later, it was found that the derivative 2 could undergo a deacetylation reaction when treated with an excess of SnCl_4_ [24] to give the starting compound (**1**). Interestingly, this deacetylation process was found to be regio- and chemo-selective, since no reaction occurred with the regioisomer **3** and the presence of *N*-acetyl or *O*-acetyl was not affected by this reaction. Finally, the presence of SnCl_4_ was required as no reaction was observed with alternative Lewis acids (AlCl_3_, SnCl_2_, ZnCl_2_) [23]. The access to derivatives **4** and **5** is easy and straightforward. The only drawback of the transformation is the use of tin(IV) salt that is reputed to be toxic.

## 3. Synthesis of 5- and 7-Aminoflavones

The naturally occurring flavones bearing the benzo-γ-pyrone structure are known for their versatile health benefits. Being polyhydroxylated, the hydroxyl groups in flavones mediate their antioxidant effects by scavenging free radicals and/or by metal chelation. Henceforth, it is now well established that the health-promoting properties of flavonoids originate from their high antioxidant capacity. In this context, flavonoids are recommended for their protective effects against chronic diseases including cancer, cardiovascular, and age-related disorders. During the Covid-19 world pandemic, flavonoids were among the most investigated and the most relevant natural products tested against the inflammatory storm caused by SARS-CoV-2 [25,26]. In the drug discovery domain, the scaffolds and the substitution patterns of naturally occurring flavones have been served as sources of inspiration for the design and development of flavones-derived drug candidates [27,28].

Among the structural modifications operated on flavonoids, the introduction of amino groups at the flavone scaffold was one of the most frequent [29,30,31,32]. For example, flavone derivatives bearing amino groups were studied as anticancer compounds [33,34,35,36]. Among them, flavopiridol was the first cyclin-dependent kinase inhibitor to enter human clinical trials [36]. Another aminoflavone, known under the name Alvocidib (AFP 464, NSC710464), has been developed by the National Cancer Institute (NCI) investigational drug program and has entered clinical trials as a promising antitumor drug candidate for the treatment of acute myeloid leukemia [37,38,39,40]. This drug acts against estrogen-positive breast cancer (ER+) with a unique mechanism of action involving the activation of the aryl hydrocarbon receptor (AhR) signaling pathway [41].

As discussed above, whatever the origin of naturally occurring flavones, the most frequent structural feature in common is a hydroxy or a methoxy group at the 5 and 7 positions. Hence, amino groups are usually introduced into the flavone scaffold on the A-ring and especially at C5 and C7, where hydroxy groups were replaced by amino groups.

**Scheme 2:**
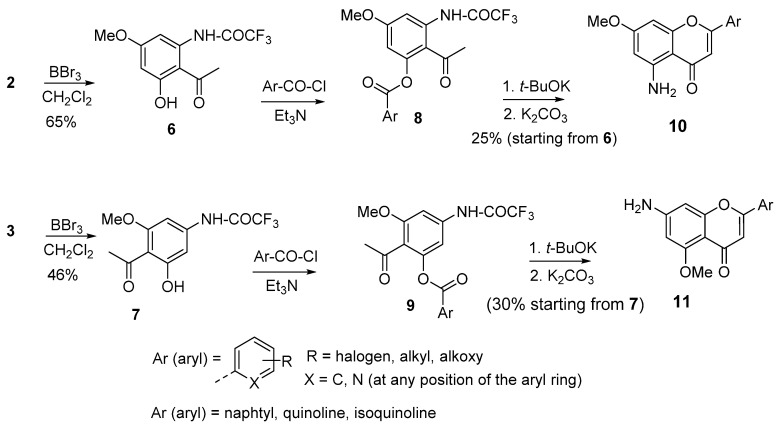
Synthesis of 5- and 7-aminoflavones.

Classically, the synthesis of simple aminoflavones can be achieved by starting either from reagents already bearing either amino groups or nitro groups. In the latter case, a nitro 2-hydroxyacetophenone reacted with a benzoyl chloride to give the corresponding diketone intermediate that is cyclized in acidic conditions to provide the corresponding nitroflavone. Finally, the nitro group is reduced either by catalytic hydrogenation or by using metal salts to give the aminoflavone [42,43]. Unfortunately, in the latter case, the reduction conditions might be incompatible with the presence of sensitive functionalities. Hence, the prospect for any alternative method to access such compounds is of interest. In this regard, the amino dimethoxyacetophenones presented in Scheme 1 offer a simple way to obtain 5-aminoflavones and 7-aminoflavones (Scheme 2). To this end, the derivatives **2** and **3**, shown in Scheme 1, were subjected to a selective demethylation reaction with diluted boron tribromide (BBr_3_) to provide the derivatives **6** and **7**. Boron tribromide demethylation is favored at the position 5; higher concentrations and a longer reaction time are needed for a possible demethylation process at the position 7 [44]. The acetophenone derivatives **6** and **7** were esterified with aroyl chloride derivatives to provide the corresponding esters **8** and **9**, which were not purified and directly cyclized upon treatment with a non-nucleophilic base such as potassium *tert*-butoxide. In cases where the desired aroyl chloride is not available, the corresponding aryl carboxylic acid can be used in the presence of a peptide coupling agent. Finally, the amino groups were deprotected by means of potassium carbonate to afford the aminoflavones **10** and **11** with acceptable mean yields, starting from **2** and **3**. The method offers the advantage of being applicable to diverse derivatives bearing a large variety of substituents on the B-ring, including halogens, alkoxides, alkyls, heterocycles, and fused cycles. It should be noted that the methoxy groups of **10** and **11** can be deprotected to obtain the corresponding aminohydroxyflavones. The only limitation of the method is the moderate yield regarding the transformation of esters **8** and **9** to the final compounds.

## 4. Synthesis of 2-Aryl-4-quinolones (Azaflavones)

2-Aryl-4-quinolones and flavones are very close analogs as they share very similar scaffolds. From the drug design point of view, it is obvious that 2-aryl-4-quinolones can be studied as drug candidates in the same pharmacological and therapeutic areas as those of flavones. During the last three decades, they have attracted extensive investigations as lead compounds for the development of drug candidates, especially as anticancer agents. K.-H. Lee and colleagues have pioneered the investigations of this class of compounds’ anticancer potential [45,46,47]. Their main mechanism of action was believed to involve the inhibition of tubulin polymerization, classifying them as anti-mitotic agents. Besides their cytotoxic activity, they were also known for their ability to prevent serotonin-induced increases in endothelial albumin permeability [48] as ligands of the neurotransmitter gamma-aminobutyric acid (GABA) receptors [49] and as xanthine oxidase inhibitors [50].

**Scheme 3:**
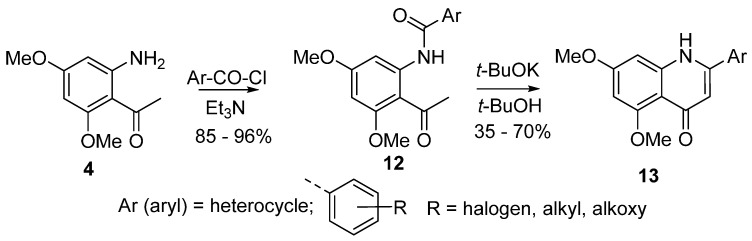
Synthesis of 2-aryl-4-quinolones starting from aminodimethoxyacetophenones.

The synthetic access to 2-aryl-4-quinolones can be achieved through two main methods. The first starts from *ortho*-amino acetophenones, which reacted with an aroyl chloride or its corresponding arylcarboxylic acid to provide the corresponding amides, followed by their cyclization [51,52]. The second method is accomplished via the palladium-catalyzed carbonylation of *O*-iodophenols and aniline in the presence of alkynes [53]. As highlighted before, the substitution pattern of flavones frequently involves a hydroxylation step and/or a methoxylation process at the 5 and 7 positions (Figure 1). Therefore, to obtain 2-aryl-4-quinolones bearing hydroxy or methoxy groups at C5 and C7, the scaffold 4 can be efficiently used, as shown in Scheme 3 [54]. To this end, the reaction of **4** with diverse aroyl chlorides in mild conditions provides the corresponding amides **12** with high yields. The final step is the cyclization of **12** with bases such as potassium *ter*-butoxide to afford the expected 2-aryl-4-quinolones **13**. The synthesis is straightforward (except in a few cases where the yield is low, 35%) and compatible with a variety of substituents that can be present at the aryl moiety.

**Scheme 4:**
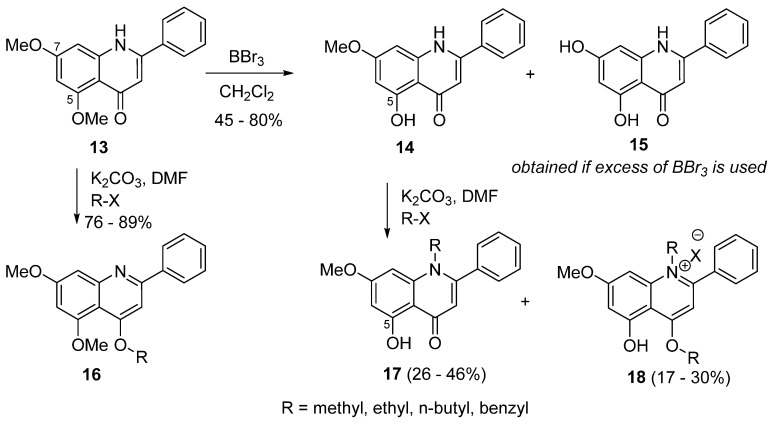
Access to chemical diversity through the alkylation of 4-quinolones.

2-Aryl-4-quinolones (**13**), shown in Scheme 3, can be transformed into a wide range of derivatives (Scheme 4). Hence, the two methoxy groups can be partially or fully deprotected to form the analogs **14** and **15**, respectively. For diversity purposes, the alkylation of **13** and **14** was reported and presented some interesting reactivity aspects. It was found that **13** and **14** behave differently according to alkylation conditions (alkyl, halide, base). In the last condition, the analogs **13** provided principally *O*-alkylated quinolines **16**, whereas the analogs **14** produced *N*-alkylquinolones **17** and dialkyl quinolines **18** [55]. The chemoselectivity of alkylation (*N* versus *O* alkylation) of **13** and **14** can be explained as follows. In the case of **13**, the amino group is deprotonated and isomerized to give the aryloxide derivative that can be alkylated. In this case, the aromatic characteristic of the final compound (**16**) is considered the driving force of the reaction. Regarding the alkylation of **14**, the presence of the hydroxyl group at position 5 may attenuate the nucleophilic property of the oxygen (through an intramolecular hydrogen bonding) following the deprotonation of the amino group. Although the yields are moderate to low, the method offers the advantage of providing molecular diversity.

## 5. Synthesis of 3-Aryl-2-quinolones

**Figure 2:**
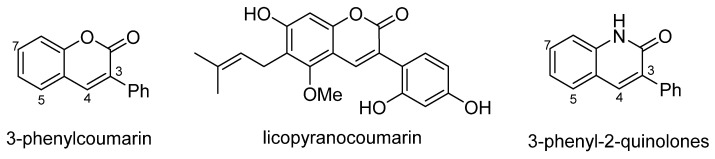
Structural analogy between 3-phenylcoumarin and 3-phenyl-2-quinolone and the structure of a natural coumarin.

3-Aryl-2-quinolones are analogs of the naturally occurring 3-phenylcoumarins (Figure 2). Coumarins display a broad range of biological activities, including antibacterial effects and the inhibition of monoamine oxidase (MAO) and the HIV replication [56,57,58]. Structural modifications of 3-phenylcoumarins were investigated in the frame of ligand-based drug discovery processes. One of the targeted structural modifications of phenylcoumarins is the substitution of the pyrane ring’s oxygen atom with a nitrogen, giving 3-phenyl-2-quinolones (Figure 2). 2-Quinolones are known for a broad spectrum of medicinal applications as drugs used in respiratory diseases, antiulcer agents, and as antiparasitic drugs [59]. Among them, 3-aryl-2-quinolones were reported as modulators of the efflux pumps involved in multi-resistant Staphylococcus aureus bacteria [60] and as anticancer agents [61].

**Scheme 5:**
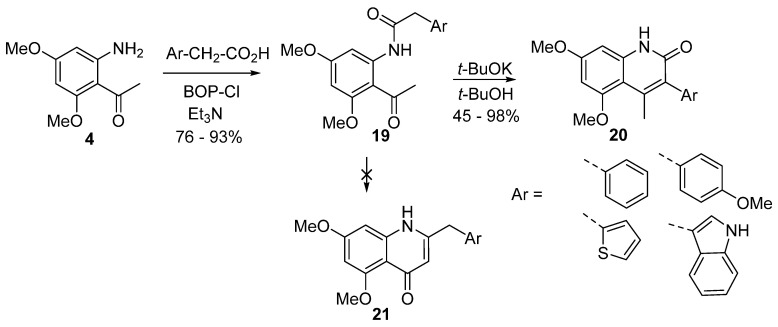
Synthesis of 3-aryl-2-quinolones.

The classical method to prepare 3-aryl-2-quinolones involves the Pd-catalyzed annulation of *o*-halo-substituted benzaldehydes with benzylamides [62]. With the aim of proposing synthetic methods that avoid the use of Pd-catalysis, to obtain 3-aryl-2-quinolones with substitution patterns similar to those found in naturally occurring coumarins, the derivative 4 (shown in Scheme 2) was reported as an ideal starting block towards the synthesis of the title compound (Scheme 5). The coupling of **4** with arylacetic acids in the presence of bis(2-oxo-3-oxazolidinyl)phosphinic chloride (BOP-Cl) affords the expected amides **19**, which, upon treatment with *t*-BuOK, afford the 4-methyl-3-aryl-2-quinolones **20** with medium to excellent yields. Throughout this sequence, the formation of other expected compounds **21** (Scheme 5) was not observed. The formation of **20** rather than that of **21** can be explained by the deprotonation preference that takes place at the benzylic carbon of **19** rather than at the acetyl moiety. This soft method can be applied to synthesize a wide range of analogs bearing different aryl groups.

## 6. Synthesis of Epoxychalcones

**Scheme 6:**
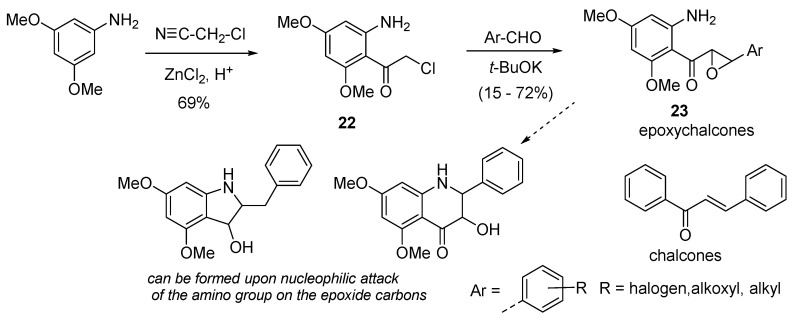
Synthesis of epoxychalcones.

A halogenated version of **4** was reported and used in the synthesis of a variety of bioactive compounds (Scheme 6). The 2′-Amino-4′,6′-dimethoxy-α-chloroacetophenone (**22**) was synthetized at a scalable level in one step starting from 3,5-aminodimethoxyaniline and chloroacetonitrile [63,64]. The Darzens reaction of **22** with aryl aldehydes in the presence of bulky and non-nucleophilic bases such as *t*-BuOK provides the epoxychalcones **23**, which are analogs of naturally occurring chalcones (Scheme 6). It is noteworthy that epoxychalcones are stable at room temperature and can be conserved for long periods. However, it was reported that, in basic media, possible cyclization through the nucleophilic attack of the amino group on the epoxide carbons might lead to the opening of their ring [64]. The epoxides **23** are analogs of the naturally occurring chalcones, which are precursors of flavonoids. Like flavonoids, chalcones have been extensively investigated as bioactive compounds in fighting against a broad range of diseases, including cancer, neurodegenerative diseases, malaria, and cardiovascular diseases [65,66]. In this context, epoxychalcones classically prepared through chalcone epoxidation by using oxidizing agents [67,68] were studied as bioactive structural chalcone analogs [68,69]. In summary, the method described above and shown in Scheme 6 can generate diversity by using diverse aryl aldehydes which are very abundant. The only drawback is the low yield of the synthesis of some derivatives of the aryl aldehyde used.

## 7. Synthesis of Azaaurones

**Scheme 7:**
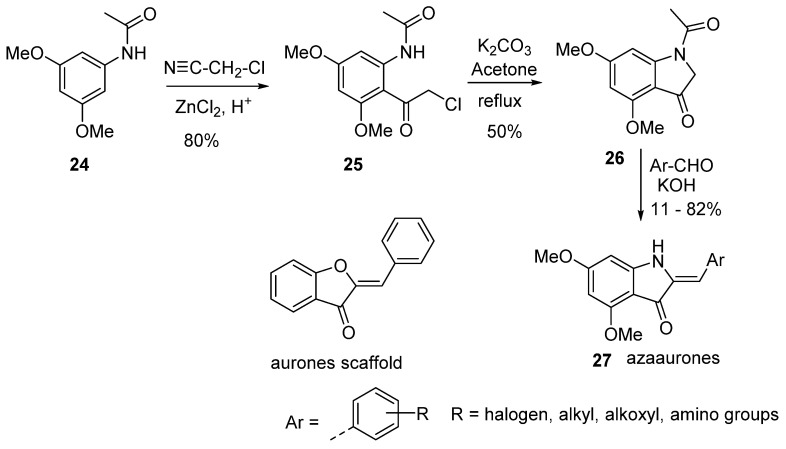
Synthesis of azaaurones.

Aurones represent a subclass of flavonoids. They can be found in fruits and flowers and, for a long time, were only considered plant pigments. The key enzymes involved in the biosynthesis of aurones were studied and led to very interesting bioengineered plants in which colored flowers were generated using aurones’ biosynthetic pathways [70]. The interest of aurones in the field of drug discovery was first reported in the late 1990s and has since been growing exponentially in major life-threatening diseases [71]. Chemical modifications of aurones with the aim of discovering drug candidates, as expected, involve the substitution of aurones’ intracyclic oxygen atom with a nitrogen (turning aurones to azaaurones). In this context, azaaurones were investigated as anticancer agents [72] and antibacterial and antiparasitic molecules [73,74,75]. In addition to their interest in the field of medicinal chemistry, azaaurones are used as key models in the development of novel organic synthesis methods [76,77]. Hence, the determination of short and effective synthetic routes to obtainazaaurones would be of high interest. To this end, the halogenated aminodimethoxyacetophenone was found to be the key starting block (Scheme 7). The acetamide derivative **24** that is quantitatively prepared from the commercially available 3,5-dimethoxyaniline was obtained. This derivative reacted with chloroacetonitrile in the presence of zinc chloride followed by acidic hydrolysis, affording the derivative **25** as the only regioisomer. Upon treatment with a base such as potassium carbonate, the compound **25** underwent a cyclisation step to afford *N*-acetyldihydroindolyl-3-one (**26**) [74]. The condensation of **26** with arylaldehydes in the presence of KOH afforded the azaaurones **27** together with the removal of the acetyl group. It was reported that the extracyclic double bond adopted the same configuration as found in naturally occurring aurones [76,77]. Owing to the therapeutic potential of azaaurones, the present method offers several advantages such as the production of **26** in large quantities and and chemical diversity.

## 8. Potential Access to Eight-Membered *N*-Heterocyclic Ring Systems (Azocanes)

**Figure 3:**
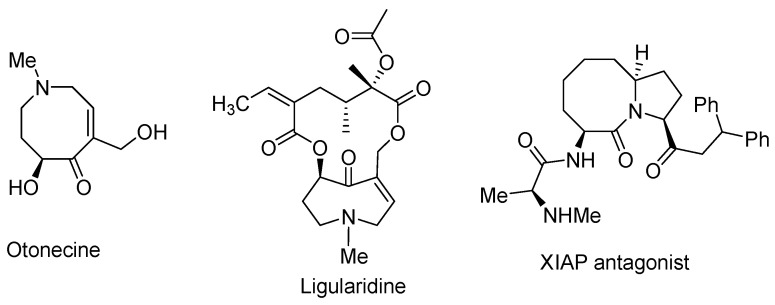
Naturally occurring compounds containing the azocane ring.

Compounds containing eight-membered *N*-heterocyclic ring systems (azacyclooctane) are known under the name azocanes. Natural products bearing such heterocyclic systems occur in many plants, with otonecine being perhaps the most common representative (Figure 3) [78]. Several compounds bearing an azocane cycle derived from otonecine, called otonecine-type macrocyclic pyrrolizidine alkaloids, were reported [79]. The latter class of compounds exhibits high genotoxicty, hepatotoxicity, cytotoxicity, and neurotoxicity as evidenced by in vitro and in vivo studies [80]. Besides their threat to humans, they also pose serious threats to livestock [81]. Despite their toxicity, macrocyclic pyrrolizidine alkaloids have been investigated in therapy. For example, the design and development of azocane-based compounds as non-peptide antagonists of the central apoptosis regulator XIAP were reported (Figure 3) [82]. Interestingly, azocanes were also reported as potent dual NK1 receptor antagonist-serotonin re-uptake transporter inhibitors for the treatment of depression [82].

**Scheme 8:**
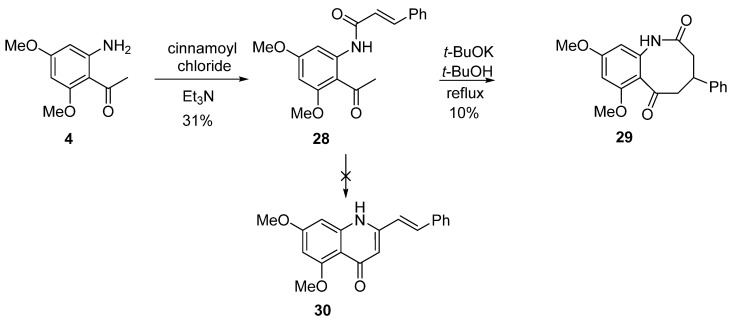
Synthesis of highly functionalized azocanes.

The synthetic methods of functionalized eight-membered *N*-heterocyclic ring systems are rare compared to those of eight-membered carbocycles. The few reported methods involve long sequences, notably if highly functionalized azocanes are targeted or the use of metal catalysis such as rhodium is needed [83,84]. Therefore, any straightforward strategy leading to functionalized azocanes would be appreciated. In this regard, the aminoacetophenone **4** described previously can be used as a key starting block for the synthesis of highly functionalized azocane derivatives (Scheme 8). The treatment of **4** with cinnamoyl chloride in the presence of triethylamine led to the formation of the expected amide **28**. The treatment of **28** with a base (in this case, *t*-BuOK) provided the azocane derivative **29** without formation of the expectable 4-quinolone **30** (Scheme 8).

**Scheme 9:**
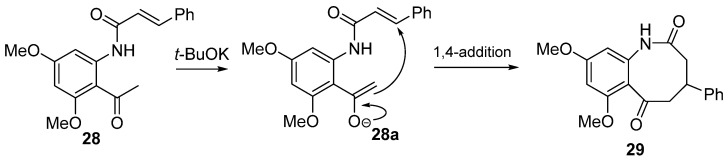
Mechanism of formation of the benzoazocane **29**.

The mechanism explaining the formation of **29** is presented below (Scheme 9). After the deprotonation of **28**, the corresponding enolate **28a** undergoes a 1,4-addition step to yield the benzoazocane **29**. Although **29** was the only azocane that was produced by this method (unpublished results) and with a modest yield, it is still interesting compared to existing options. Hence, this method deserves to be investigated using analogs of cinnamic acid and by varying the substitution patterns at the acetophenone moiety.

## 9. Conclusions

In the fields of drug discovery, chemical genetics, chemical probes, and chemical biology, the knowledge of small molecules and novel molecular entities has never been more important. In order to meet this demand, organic chemists are striving to find new synthetic strategies and approaches to produce more and more drug-like molecules. In this context, the application of diversity-oriented synthesis (DOS) to natural products can be considered a valuable option as it speeds up the identification of novel compounds starting from biologically relevant counterparts.

The purpose of this review was to report the potential of amino dimethoxyacetophenones as starting blocks to obtain different classes of heterocyclic derivatives that are analogs of naturally occurring molecules. The starting blocks reported in this article are easily prepared at a multigram scale and in stable forms. The only drawback linked to their synthesis is the use of toxic Lewis acids such as tin chloride. The reported methods involving these starting blocks are applicable to a wide range of compounds bearing variable substituents including aminoflavones, 4-quinolones, 2-quinolones, epoxychalcones, azaaurones, and azocanes. Following short and easy syntheses, chemical libraries of highly functionalized heterocyclic compounds were reported. The generated compounds were studied as hit, lead, and drug candidates against diverse life-threatening diseases including cancer, neurodegenerative diseases, bacterial infections, and malaria. The required chemistry lends itself well to parallel and solid phase syntheses, offering possibilities to enhance the chemical diversity profile of the expected molecules.

## Data Availability

Data is contained within the article.
